# Comment on “Evaluation of Complication Rates after Breast Surgery Using Acellular Dermal Matrix: Median Follow-Up of Three Years”

**DOI:** 10.1155/2018/5231565

**Published:** 2018-06-05

**Authors:** S. H. Heisterkamp

**Affiliations:** ^1^BioStat-Plus B.V., Mariaplaats 32E, 3511 LL Utrecht, Netherlands; ^2^University of Groningen, Groningen, Netherlands

 I read with great interest the article by Paprottka et al. [1] which presented the results of a study on complications after breast surgery. The publication of such a study is important for both surgeons and patients, in order to make an informed decision on the expected outcomes of such an invasive surgery. In particular, patients should be aware of the risks involved in such an intervention. Therefore it is of the utmost importance that the design of such a study is correct and that the conclusions are justified by the data. I am concerned with both of these aspects of the study, as well as fundamental dissimilarities in the devices as well as the patient cohorts.

## 1. Device Dissimilarities

To start with the former, the title of the study states that the research is a comparison of acellular dermal matrices; this, however, is not the case and will set the reader on the wrong foot. While two of the products (Epiflex and Stratice) are dermal matrices, Tutomesh is derived from bovine* pericardium* which differs fundamentally from the other two. Although both dermis and pericardium consist of dense, irregular, connective tissue, the proteins present in the tissue vary greatly. As one example, bovine pericardium has a much lower elastin content (2.98%) relative to ADM products (5–7%) [2, 3]. Therefore, the essential dissimilarity between the matrices may make comparisons difficult.

## 2. Group Dissimilarities

In addition to the tissue dissimilarities, there is also the question of whether the patients in each group are comparable. In Table 1, the average age of the patients is presented, yet I would have expected an ANOVA to compare the mean age of the groups but the authors have omitted this important step in analyzing the patient cohorts. Therefore, all the reader can go by is the apparent age presented, in which case the Epiflex patients seem to be much younger than the other cohorts. Previous research has shown higher seroma development and infection in older patients [4]. Also, there is a greater risk of capsular contracture in older patients [5].

Even more concerning are differences in numbers between the indications for implantation. The Epiflex patients were mostly seen for aesthetic indications, with 70% of Epiflex patients being treated for a primary augmentation in contrast to 30% of those treated with Tutomesh. In other words, the majority of Tutomesh patients (11 out of 16 or 70%) were seen for medical indications, either oncologic or “other.” When we assessed the distribution of patients as either medical or aesthetic indication, Fisher's exact test (see the subsection on statistical analysis) showed a significant difference in the distribution of patients (*p* = 0.03). There were significantly fewer Epiflex patients seen with an indication of oncology. This is directly relevant to the outcomes measured, as radiotherapy or chemotherapy has a notable adverse impact on ADM remodeling [6] and, as a whole, patients seen for medical indications are certainly in a less healthy condition. Specific to that, the authors failed to define what the other indications were. With only four patients in this group, a simple list would have sufficed. Therefore, it can be concluded that the group of patients treated with the different implants are not comparable.

## 3. Statistical Analysis

With respect to statistical analysis of the data, I am of the opinion that the analysis performed by the authors is not valid. A correct statistical analysis shows that the (implicit) conclusion that all three products differ with regard to the percentage of complications is not supported by the data. Furthermore the study design itself is open to discussion and consequently its statistical analysis.

First of all, the authors claim to have used the Mantel-Haenszel method, both for comparing the frequencies and for performing a trend test. The former is completely impossible, and the latter is incorrect. The Mantel-Haenszel method is designed to combine the Chi-square results of several independent 2-by-2 tables. There is only one 3-by-2 table, and any subdivision into other 2-by-2 tables, for example, Epiflex compared to the other treatments and Tutomesh with the other two, invalidates the use of the Mantel-Haenszel method. Furthermore the test on trend assumes that there is a predefined ordering of the three treatments. There is no such a priori ordering. These two severe mistakes are in fact enough to dismiss the statistical results of the study.

Our claim that a correct statistical analysis indeed questions the conclusions of the authors is based on a generally known property of any Chi-square statistics used in cross-tables. The use of a Chi-square test is invalid for tables with small frequencies. The frequency of the complications is far too small to use the Chi-square approximation. A well-known rule of thumb is that the* expected* frequencies under the null hypothesis of no difference should be at least *n* = 5. The expected frequencies of complications under H_0_ are computed as 9/52 times the marginal totals of each product, that is, 15, 21, and 16. Applying this rule the expected number of complications is 2.6, 3.6, and 2.8, respectively, all of which smaller than 5. The effect of these small numbers is that the value of the Chi-square will be artificially inflated, hence yielding a* too small p* value, which may lead to an incorrect decision with regard to “significance.”

For a robust analysis of tables with small numbers Fisher's exact test is to be used. This test was originally proposed by Fisher [7] for 2-by-2 tables but thanks to better algorithms and more computing-power number of both rows and columns may be larger than 2 and enables the computation of the exact *p* value in a limited time. It should be noted that for large expected values this test is equivalent to the Chi-square approximation.

Fisher's exact test yields a *p* value of 0.1904, which is far from significant using a threshold of 0.05. Effectively, this means that the data of this study are not supportive for the implicit conclusion that the probabilities of complications differ for the three products. In Table 1 this is illustrated by presenting the exact 95% confidence limits of the proportions of complications for each study under the null hypothesis. All confidence intervals of a product have a considerable overlap with each of the other products. Note that confidence intervals from a binomial distribution need not be symmetrical.

## 4. Study Design

Finally, the fact is that the statistical analysis—even if a Fisher's exact test had been used—is not compatible with the study design. The statistical analysis from the article as the one presented above supposes that all samples are independently acquired. However it was reported that 41 patients had undergone surgery whereas 52 breasts (sampling units) were reconstructed. This means that some patients had undergone reconstructions for two breasts, or that some breasts had two surgeries. The latter is suggested by Table 2 of the paper—last column with heading: “Secondary augmentation* after* (my italics) capsular contracture”). From that we infer that 6 breasts had been reconstructed twice. If this interpretation is correct, then the total independent samples should be decreased to 46, which still leaves at least 5 patients with surgical reconstruction on two breasts. As a complication with a reconstruction of one breast may indirectly influence the other one of the same patient, it is not guaranteed that the observations are independent. In one way or another, the total number of samples used in the statistical analysis should have been based on the results of 41 breast reconstructions from 41 patients. As this is not the case, it is even harder to draw conclusions from these (retrospective) data given the small numbers. The correct study design should have anyway excluded 6 sampling units which had surgery twice and should also have randomly included only one of the two breasts in case a patient had both breasts reconstructed. The design of a retrospective study as proposed above mimics the procedure commonly used in a prospective study to guarantee independently acquired sampling units.

As a last minor remark, both in the title of the article and in the text, the word “median” follow-up is used, whereas in Table 1 the heading for follow-up time indicates average (Avg.). For age, the word average is also used but from the text it was not clear whether this was correct or not.

## 5. Overall Conclusion

We conclude that the implants used are fundamentally different, the patients groups are not comparable with respect to age and indications for surgery, and the statistical tests used by the authors are invalid. Moreover the design of the retrospective study is flawed. Even if we accept the design “as is,” the use of an appropriate test shows that the data provided do not support the conclusion of the authors. All in all, the conclusions of the authors that there are differences in the probability of complications between the three treatments are to be dismissed.

Finally, I hope that the above remarks with respect to the statistical analysis will result in a revision of the article, in the sense that the data do not support evidence that the proportions of complications differ among products. I also hope that the remark on the design of the study positively contributes to the design of a further prospective study, should the authors undertake such an effort.

## Figures and Tables

**Table 1:** Estimated probability of complications and 95% confidence intervals under the H_0_ hypothesis of equal probability. The expected frequencies do not add to 9 due to rounding to the first decimal.

ADM	Product	Observed percentage of complications	95% confidence interval under H_0_	Expected frequencies under H_0_
HADM	Epiflex/DIZG	7%	0%–40%	2.6
PADM	Strattice/LifeCell	14%	5%–33%	3.6
BADM	Tutomesh/RTI Surgical	31%	0%–38%	2.8
	Overall	17%	8%–27%	9

## References

[1] Paprottka F. J., Krezdorn N., Sorg H. (2017). Evaluation of Complication Rates after Breast Surgery Using Acellular Dermal Matrix: Median Follow-Up of Three Years. *Plastic Surgery International*.

[2] Gaertner W. B., Bonsack M. E., Delaney J. P. (2007). Experimental evaluation of four biologic prostheses for ventral hernia repair. *Journal of Gastrointestinal Surgery*.

[3] Mofid M. M., Meininger M. S., Lacey M. S. (2012). Veritas® bovine pericardium for immediate breast reconstruction: A xenograft alternative to acellular dermal matrix products. *European Journal of Plastic Surgery*.

[4] Song D., Slater K., Papsdorf M. (2016). Autologous Breast Reconstruction in Women Older Than 65 Years Versus Women Younger Than 65 Years: A Multi-Center Analysis. *Annals of Plastic Surgery*.

[5] McGuire P., Reisman N. R., Murphy D. K. (2017). Risk Factor Analysis for Capsular Contracture, Malposition, and Late Seroma in Subjects Receiving Natrelle 410 Form-Stable Silicone Breast Implants. *Plastic and Reconstructive Surgery*.

[6] Myckatyn T. M., Cavallo J. A., Sharma K. (2015). The impact of chemotherapy and radiation therapy on the remodeling of acellular dermal matrices in staged, prosthetic breast reconstruction. *Plastic and Reconstructive Surgery*.

[7] Fisher R. A. (1935). The logic of inductive inference. *Journal of the Royal Statistical Society*.

